# Ciliate Environmental Diversity Can Be Underestimated by the V4 Region of SSU rDNA: Insights from Species Delimitation and Multilocus Phylogeny of *Pseudokeronopsis* (Protist, Ciliophora)

**DOI:** 10.3390/microorganisms7110493

**Published:** 2019-10-26

**Authors:** Zifeng Zhan, Ju Li, Kuidong Xu

**Affiliations:** 1 Laboratory of Marine Organism Taxonomy and Phylogeny, Institute of Oceanology, Chinese Academy of Sciences, Qingdao 266071, China; zzhan@qdio.ac.cn (Z.Z.); liju19891007@163.com (J.L.); 2 Laboratory for Marine Biology and Biotechnology, Pilot National Laboratory for Marine Science and Technology (Qingdao), Qingdao 266071, China; 3 Center for Ocean Mega-Science, Chinese Academy of Sciences, Qingdao 266071, China; 4 University of Chinese Academy of Sciences, Beijing 100049, China

**Keywords:** metabarcode, DNA barcoding, cryptic species, secondary structure, ITS1-5.8S-ITS2, mitochondrial COI, rDNA

## Abstract

Metabarcoding and high-throughput sequencing methods have greatly improved our understanding of protist diversity. Although the V4 region of small subunit ribosomal DNA (SSU-V4 rDNA) is the most widely used marker in DNA metabarcoding of eukaryotic microorganisms, doubts have recently been raised about its suitability. Here, using the widely distributed ciliate genus *Pseudokeronopsis* as an example, we assessed the potential of SSU-V4 rDNA and four other nuclear and mitochondrial markers for species delimitation and phylogenetic reconstruction. Our studies revealed that SSU-V4 rDNA is too conservative to distinguish species, and a threshold of 97% and 99% sequence similarity detected only one and three OTUs, respectively, from seven species. On the basis of the comparative analysis of the present and previously published data, we proposed the multilocus marker including the nuclear 5.8S rDNA combining the internal transcribed spacer regions (ITS1-5.8S-ITS2) and the hypervariable D2 region of large subunit rDNA (LSU-D2) as an ideal barcode rather than the mitochondrial cytochrome c oxidase subunit 1 gene, and the ITS1-5.8S-ITS2 as a candidate metabarcoding marker for ciliates. Furthermore, the compensating base change and tree-based criteria of ITS2 and LSU-D2 were useful in complementing the DNA barcoding and metabarcoding methods by giving second structure and phylogenetic evidence.

## 1. Introduction

DNA barcoding is an exciting tool to assess biodiversity, and the mitochondrial cytochrome c oxidase subunit 1 (COI) gene as a barcode has been successfully used in many animal taxa [1,2,3]. In the case of eukaryotic microorganisms, the Protist Working Group proposed a two-step barcoding pipeline for eukaryotic microorganisms: first, the hypervariable V4 region of small subunit ribosomal DNA (SSU-V4 rDNA) is applied to assign organisms to a high-ranking taxonomic group; second, a group-specific barcode is used for species-level assignments [4]. Many microbial eukaryotic groups, however, still lack a suitable barcode [5]. At the current stage, small subunit ribosomal DNA (SSU rDNA) is still the primary marker used to identify species and to examine their phylogenetic positions [6,7]. On the basis of the large sequence database, the SSU-V4 rDNA has been widely chosen as a metabarcode for high-throughput sequencing analyses to assess the environmental diversity of the eukaryotic microorganisms [8,9,10]. However, both the partial and complete SSU rDNA have been questioned as being too conserved to distinguish closely related species and to uncover cryptic species [9,11,12,13,14,15]. Thus, there is a pressing need to test other gene markers as suitable barcodes and metabarcodes.

*Pseudokeronopsis* Borror and Wicklow, 1983 is a conspicuous ciliate genus, in which most species have a colourful body and occur at high frequencies in marine or limnetic habitats worldwide [7,12,16,17,18,19,20]. Nowadays, there is growing interest in using *Pseudokeronopsis* in various research fields including chemical defense mechanisms, actin evolution, and morphogenesis [18,21,22,23]. *Pseudokeronopsids* are distinguished mainly by the morphological combination of ciliary pattern, cortical granules, and contractile vacuole [12,16,19]. However, *Pseudokeronopsis* is one of the most difficult genera of ciliates for species discrimination due to its high interspecific similarities [12,16,19,20]. Many populations were often misidentified as certain “well-known” species, for example, the type species *Pseudokeronopsis rubra* and *Pseudokeronopsis carnea* [12,16,19]. Consequently, many *Pseudokeronopsis* sequences in GenBank are dubious, and even the correct sequences of the type species are awaiting being submitted [9]. On the other hand, the inner phylogenetic relationships are still relatively unknown due to the incomplete or inaccurate morphological and molecular data [12,16,19,20,24]. *Pseudokeronopsis* frequently occurs in marine habitats, and 7 of 12 known species could be sampled in the same location, that being the coastal area of Qingdao, China (Table 1). Hence, the ciliate genus *Pseudokeronopsis* is an ideal taxon that can be utilized to test the potential of different markers for species delimitation, environmental diversity estimation, and phylogenetic reconstruction of the eukaryotic microorganisms. 

No universal barcode has been accepted for the species delimitation of ciliates [14]. The mitochondrial COI and cytochrome b and the nuclear 5.8S rDNA combining the internal transcribed spacer regions (ITS1-5.8S-ITS2) and the hypervariable D1-D2 region of large subunit ribosomal DNA (LSU rDNA) have showed varying degrees of barcoding potential [5,13,14,15,25,26,27,28,29]. Among them, COI is most frequently tested as a candidate barcode, providing much better resolution at the intraspecific level than that of the nuclear genes [13,14,25]. Recently, the D1-D2 region of LSU (LSU-D1-D2) rDNA was proposed as a good candidate barcode for ciliates due to the fact that it shows clear barcoding gaps within *Euplotes*, *Paramecium,* and Tintinnida [5,9,15]. Due to the ITS1 and ITS2 regions having shown much higher rates of evolutionary changes than the code regions of the ribosomal subunits, the ITS1-5.8S-ITS2 and ITS2 regions are also popular markers for the ciliate identification and investigation of intraspecific genetic diversity [30,31,32,33]. However, the barcoding efficiency of the mitochondrial and nuclear markers was only assessed in several oligohymenophorean genera (e.g., *Paramecium*, *Frontonia,* and *Tetrahymena*) with congeneric and conspecific sequences [15,25,34]. No such assessment has been made for other groups, for example, the most diverse ciliate class Spirotrichea. 

In the present study, we assessed the potential of the genetic distance of the ITS1-5.8S-ITS2, ITS2, LSU-D2, SSU-V4 rDNAs, and the mitochondrial COI, as well as the secondary structures of ITS2 and LSU-D2 for species discrimination of the spirotrichean genus *Pseudokeronopsis*. Subsequently, we revised all available *Pseudokeronopsis* sequences in GenBank on the basis of the barcoding analyses combined with morphological data. On the basis of the comparative analysis of the present and previously published data, we examined which markers were appropriate barcodes and metabarcodes for ciliates. Simultaneously, we compared the resolving power of the selected candidate barcodes and SSU rDNA as well as the concatenated nuclear and mitochondrial regions for the phylogenetic reconstruction. By combining the morphological and molecular data, we inferred the putative phylogenetic relationships for all known species of *Pseudokeronopsis*. The objective of this study was to shed more light on the screening of barcodes and metabarcodes for the eukaryotic microorganisms.

## 2. Materials and Methods

### 2.1. Specimen Collection and Identification

*Pseudokeronopsis rubra*, the type species of the genus, was collected in April 2017 from the Qingdao Port (36°05′17″ N, 120°19′21″ E). *Pseudokeronopsis* cf. *songi* was collected in April 2018 from the treated sewage outfall near Qingdao No. 1 Bathing Beach (36°03′25″ N, 120°20′10″ E) on the China coast of the Yellow Sea. *Pseudokeronopsis erythrina* was collected in November 2014 from the Sheyang Estuary (33°49′00″ N, 120°28′05″ E) on the China coast of the Yellow Sea. The genomic DNAs of *P. carnea*, *P. pararubra*, and *P. songi* were obtained from [12].

Cells from field samples were isolated and observed in vivo at 100–1000× magnifications using bright field and differential interference contrast microscopy. The ciliates were impregnated with protargol following [38] to reveal the ciliary and nuclear pattern for morphological identification. Terminology and systematics mainly followed [7,16]. 

### 2.2. DNA Extraction, PCR Amplification, and Sequencing

Monoclonal cultures for *Pseudokeronopsis* cf. *songi* and *P*. *erythrina*, and rough culture for *P. rubra* were established. Live cells were maintained in filtered seawater overnight, and then rinsed in filtered seawater four times. One cell of *P.* cf. *songi* and *P. rubra*, and three cells of *P*. *erythrina* were transferred into three microfuge tubes for DNA extraction using DNeasy Blood and Tissue Kit (Qiagen, Hilden, Germany), according to the manufacturer’s instructions. PCR amplification for small subunit ribosomal DNA (SSU rDNA) was conducted using primers 16S-F (5′-AACCTGGTTGATCCTGCCAGT-3′) and 16S-R (5′-TGATCCTTCTGCAGGTTCACCTAC-3′) as follows: denaturation at 98 °C for 2 min, followed by 35 cycles of denaturation at 98 °C for 10 s, annealing at 58 °C for 12 s, extension at 72 °C for 20 s, and a final extension step at 72 °C for 5 min [39]. PCR amplification for internal transcribed spacer region ITS1-5.8S-ITS2 and a 5′-end region of large subunit ribosomal DNA (ITS1-5.8S-ITS2-5′LSU rDNA) sequences was conducted using the ITS-F (5′-GTAGGTGAACCTGCGGAAGGATCATTA-3′) [40] and CILI28S-1000 (5′-CATTCGGCAGGTGAGTTGTTACACTCC-3′) [41] primers that were complementary to conserved regions and encompassed the 3′-end of the SSU rDNA (27 bp), the entire ITS1-5.8S-ITS2 region, and the complete D1-D2 and partial D3 regions of the large subunit rRNA. The amplification cycle conditions were the same with that of SSU rDNA. Mitochondrial COI gene was amplified with the primer set (CiCOIFv2: 5′-GWTGRGCKATGATYACACC-3′; CiCOIRv2: 5′-ACCATRTACATATGATGWCC-3′), with the following conditions: 2 min at 94°C followed by 40 cycles (95 °C for 15 s, 53 °C for 30 s, and 72 °C for 60 s) and a final extension at 72 °C for 5 min [27]. PCR reactions were performed using I-5 2x High-Fidelity Master Mix DNA polymerase (TP001, Beijing Tsingke Biotech Co., Ltd.). PCR purification and sequencing were performed by Tsingke (Beijing, China). 

### 2.3. Genetic Distances, Operated Taxonomic Unite Delimitation, and Phylogenetic Analyses

All the available SSU rDNA, ITS1-5.8S-ITS2, LSU rDNA, and COI sequences of *Pseudokeronopsis* spp. except for those considered as non-*Pseudokeronopsis* by [12] and the out-group species from related hypotrich genera were downloaded from GenBank (Table 1). On the basis of the morphological classification of [9], the previously sampled populations of *P. rubra* and *P. carnea* were misidentified and marked with asterisks in the tables and figures of the following analyses. These sequences and those noted as *Pseudokeronopsis* sp. or submitted without associated morphological publications were excluded from the initial statistics of intra- and interspecific distances. 

Sequences of each region were aligned independently using MAFFT v.7 with the G-INS-i and Q-INS-i (secondary structure of RNA is considered) algorithms for the COI and ribosomal regions [42]. The SSU-V4, ITS1-5.8S-ITS2, ITS2, and LSU-D2 sequence datasets were constructed according to the previous secondary structure predictions by [43,44,45]. For the barcoding analysis, genetic distances between species/populations were calculated with MEGA 6.0 [46] using the Kimura two-parameter model. Considering that 7 of 12 known *Pseudokeronopsis* species can be sampled in the same location, that is, the coastal area of Qingdao, China (Table 1), we also compared the species diversities detected by the metabarcoding, barcoding and morphological methods. Using UPARSE for the metabarcoding analysis, sequence similarities of 97% and 99% of SSU-V4 were applied to delineate operated taxonomic unites (OTUs) of *Pseudokeronopsis* [47].

Phylogenetic analyses were performed on the datasets of SSU, SSU-V4, and LSU-D2 rDNA; ITS1-5.8S-ITS2, ITS2, COI, and the concatenated SSU-ITS1-5.8S-ITS2-5′LSU rDNA; and rDNA-COI. The best-fit models, GTR + I + G for SSU-V4, LSU-D2 rDNA, ITS1-5.8S-ITS2, ITS2, COI and the concatenated regions, and TIM3 + I + G for SSU rDNA were selected with the Akaike information criterion in jModelTest2 v2.1.6 [48]. For the amino acid alignment of COI, the MtZoa + G evolutionary model was the best-fitted model selected by SMS in PhyML [49,50]. Maximum likelihood (ML) analyses were carried out using PhyML-3.1 [49] with node support from a majority-rule consensus tree of 1000 bootstrap replicates. For the ML bootstraps, we considered values <70% as low, 70–94% as moderate, and ≥95% as high, following [51]. Bayesian inference (BI) was carried out using MrBayes v3.2.6 [52]. Posterior probability was estimated by using four chains running 10,000,000 generations sampling every 1000 generations. The first 25% of sampled trees were considered burn-in trees. For the Bayesian posterior probabilities, we considered values <0.95 as low and ≥0.95 as high, following [53]. 

### 2.4. Secondary Structure Prediction

Consensus structures of *Pseudokeronopsis* ITS2 and LSU-D2 regions (Figures 3A and 5A) were predicted using the LocARNA Sever with default options (available from http://rna.informatik.uni-freiburg.de/LocARNA/Input.jsp) [54]. The consensus secondary structure of ITS2 was compared with the structural template constructed by other spirotrichean ciliates [40], manually corrected and used as a model for *Pseudokeronopsis* species. With the guidance of the model, putative secondary structures of the *Pseudokeronopsis* ITS2 molecules were constructed on RNAfold server of the ViennaRNA Web Services (available from http://rna.tbi.univie.ac.at/cgi-bin/RNAfold.cgi) [55] with constraint folding strategy. The folding results for minimum free energy (MFE) structure drawing encoding base-pair probabilities were chosen as outputs. 

The model of LSU-D2 secondary structure was not available for any ciliate group. Consequently, the structures predicted using the default values were compared to each other as well as the consensus one to establish the model. The structures of all *Pseudokeronopsis* populations except *P. carnea* JQ424836 are nearly identical with the consensus one, which was selected as the LSU-D2 model in *Pseudokeronopsis*. On the basis of the model, the secondary structure of *P. carnea* JQ424836 was reconstructed with constraint folding strategy. Once the conserved structural models of ITS2 and LSU-D2 in *Pseudokeronopsis* ciliates were established, compensating base changes (CBCs, e.g., G = C ↔ C = G, A = U or U = A) were also detected with the CBCAnalyzer [56] as implemented in 4SALE, and hemi-CBCs (compensatory change on lonely one side of a helix pairing, i.e., G = C ↔ G = U) were found manually. 

## 3. Results

### 3.1. Molecular Sequences, Genetic Distance, and Species Delimitation Analyses

A total of 28 new sequences from six species were deposited in GenBank and their accession numbers are provided in Table 1. The alignments of SSU-V4, ITS2, ITS1-5.8S-ITS2, LSU-D2, and COI had lengths of 226 bp, 199 bp, 494 bp, 247 bp, and 480 bp, respectively. The inter- and intraspecific genetic divergences of these alignments were calculated to investigate the genetic distances in *Pseudokeronopsis*. In total, seven species: *P. rubra*, *P.* cf. *songi*, *P. songi*, *P. erythrina*, *P. carnea*, *P. pararubra,* and *P. flava* were included the initial statistics of genetic distances among conspecifics and congeners. Simultaneously, sequences noted as *Pseudokeronopsis* sp. and those without associated accurate morphological publications (marked with gray shadows in Table S1–S5) were excluded from the initial statistics. Maximum genetic distances among conspecifics and minimum genetic distances among congeners were used to identify those uncertain species. 

**Figure 1:**
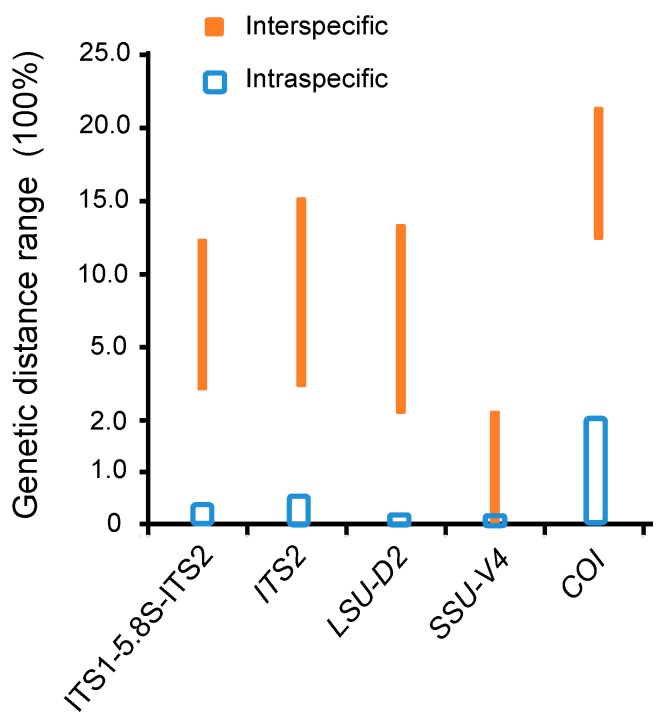
Genetic distance ranges of ITS1-5.8S-ITS2, ITS2, LSU-D2, SSU-V4, and COI within *Pseudokeronopsis*.

For the COI alignment, the genetic distances among the former six species ranged from 14.27% to 22.27%, whereas there was no genetic variation based on the initial statistics of intraspecific distances (Table S1). On the basis of the distance data and the lack of morphological information, *P. rubra* MG594873 and *P. carnea* MG594874 + MG594875 (marked as C4 and C5 in the COI trees) were regarded as two uncertain species of *Pseudokeronopsis* (Table 2). When the data of all known and uncertain species was included, the intraspecific distances of COI were in the range of 0–2.58%, whereas the interspecific ones were in the range of 13.25–22.28% (Table S1; Figure 1). 

Like the case of COI, the genetic distances among the seven species were also distinctly higher than those among conspecifics for the ITS1-5.8S-ITS2 (3.68–13.01% vs. 0–0.42%), ITS2 (6.55–16.93% vs. 0–0.52%), and LSU-D2 (2.53–17.51% vs. zero; Table S2–S4). When these cryptic and uncertain species (marked as C1, C2, and C3 in the ITS1-5.8S-ITS2 trees) were included, no change occurred in the range of the intraspecific distances, whereas the interspecific distances were slightly extended to 3.23–13.28% for ITS1-5.8S-ITS2, 3.72–16.93% for ITS2, and 2.1–17.51% for LSU-D2 (Table 2, Table S2–S4; Figure 1). 

The SSU-V4 genetic distances among the seven species ranged from zero to 2.75%, corresponding to zero to six variable sites and the sequence similarity of 97.3–100%. No intraspecific variation existed (Table S5). *Pseudokeronopsis* cf. *songi, P. songi*, the uncertain species C4, and *P. rubra* HQ228548 (C2) were even identical in the SSU-V4 sequence. 

**Figure 2:**
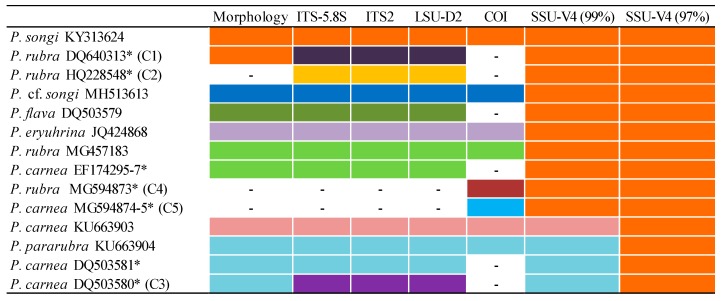
Horizontal bars show the species delimitation results inferred from the morphology and molecular markers (listed on the upper side) for *Pseudokeronopsis* species. Each color represents a species (online version in color). C1–C5 correspond to the C1–5 taxa marked in the following phylogenetic trees. Dashed line indicates no available data. *, misidentified species.

All available sequences of *Pseudokeronopsis* in GenBank were assigned to seven species (Table 1; Figure 2). The species delimitation results based on the ITS1-5.8S-ITS2, ITS2, and LSU-D2 were identical, which not only further confirmed the status of the seven species, but also uncovered the two cryptic species C1 and C3 and the uncertain species C2 (Table 2; Figure 2). The COI sequences were only available for six nominal species, and the species delimitation analysis conformed the status of the six species and distinguished the two uncertain species C4 and C5 as well (Table 2; Figure 2). In contrast, only one and three OTUs could be detected from the seven species when using the SSU-V4 sequence similarity of 97% and 99%, respectively, as a threshold (Figure 2). Because C4 and C5 had no common marker sequences comparable with C1–C3, we could not determine whether C4 and C5 belonged to part of C1–C3. Nonetheless, the species delimitation analyses indicated that the available sequences represented at least 10 *Pseudokeronopsis* species including 7 known species, 2 cryptic, and at least 1 uncertain species.

### 3.2. ITS2 and LSU-D2 Secondary Structures

**Figure 3:**
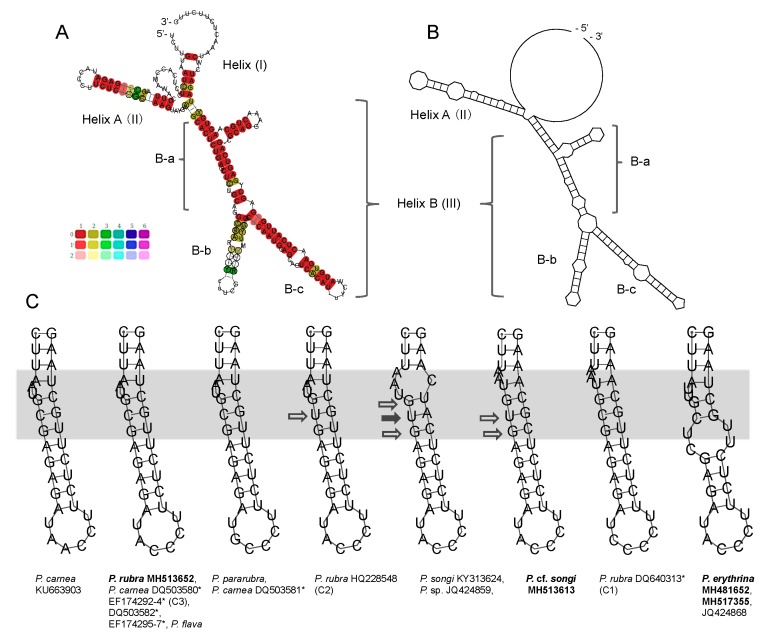
Secondary structure features of the *Pseudokeronopsis* ITS2 molecule. (**A**) Consensus secondary structure predicted using the LocARNA Sever. Color bars (0–1) indicate base-pairing probability. (**B**) Structural model for *Pseudokeronopsis*. (**C**) Helix A structures of congeners. Using the structure of *P. carnea* as a reference, compensating base changes (CBCs) are marked with filled arrows and hemi-CBCs are indicated with hollow arrows. The variable region is marked by a grey box. Newly sequenced species are in bold. *, misidentified.

**Figure 4:**
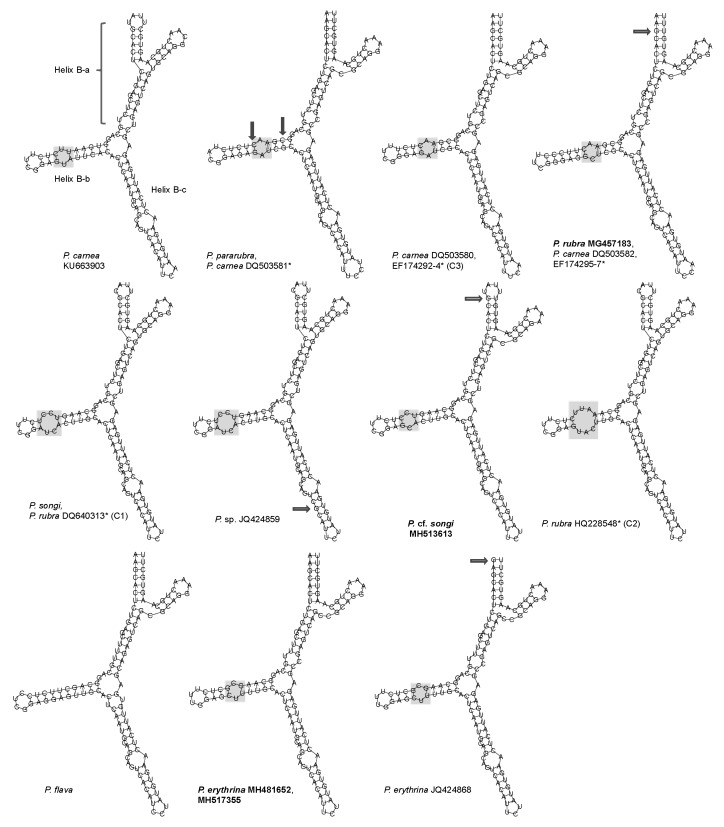
Helix B secondary structures of the *Pseudokeronopsis* ITS2 molecule. Using the structure of *P. carnea* as a reference, CBCs are marked with filled arrows and hemi-CBCs are indicated with hollow arrows. Newly sequenced species are in bold. *, misidentified.

Putative secondary structures of ITS2 transcripts are proposed in Figure 3 and Figure 4, possessing the following features: (1) an internal loop bearing two helices, (2) a conserved Helix A with a motif 5′-GAGA versus UCUC-3′ and a U-U mismatch in the terminal loop, and (3) Helix B being the longest one and containing three sub-helices (B-a,b,c). The lengths of the all the parts except for helix B were identical, and Helix A was much shorter than Helix B (32 vs. 114–117 nt; Figure S1). *Pseudokeronopsis rubra* MH513652, *P. pararubra*, *P. flava*, and *P. carnea* KU663903, DQ503580, DQ503582, and EF174292–7 shared the same structure pattern of Helix A without any CBC or hemi-CBC (Figure 3C). The CBCs, hemi-CBCs, and/or different patterns of the bugles were present within Helix A in other species of *Pseudokeronopsis* (marked with filled and hollow arrows in Figure 3C). The structures of the sub-helices B-a and c were highly similar among *Pseudokeronopsis* species, whereas the sub-helix B-b showed much variation in lengths, the positions of the bulge, and the numbers of CBC and hemi-CBA (Figure 4). The CBC analysis revealed a maximum of two CBCs between *Pseudokeronopsis* species and no CBC between the conspecifics (Table 3). 

**Figure 5:**
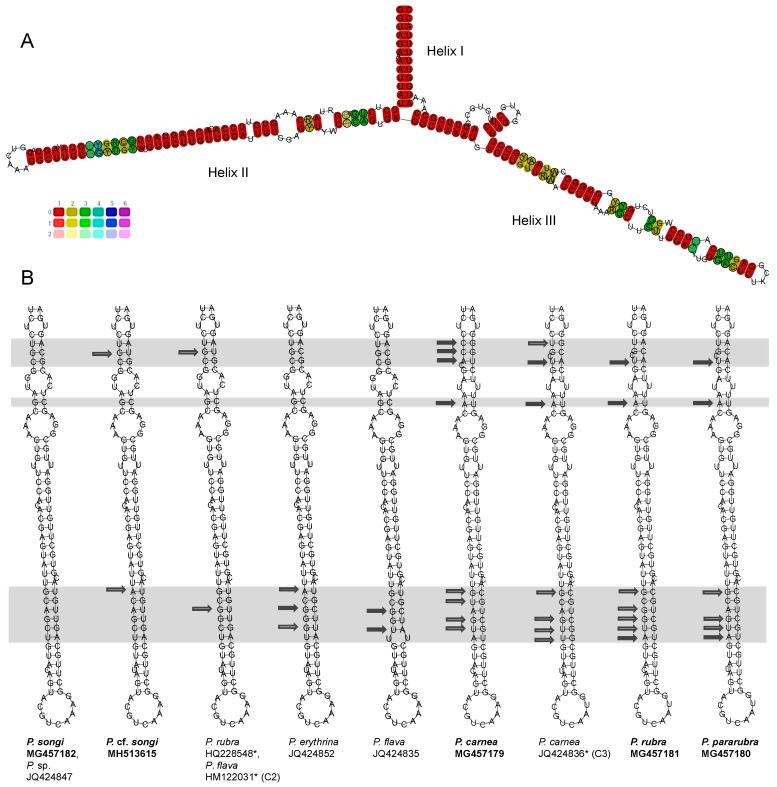
Secondary structure features of the *Pseudokeronopsis* LSU-D2 molecule. (**A**) Consensus secondary structure predicted using the LocARNA Sever. Color bars (0–1) indicate base-pairing probability. (**B**) Helix II structures of congeners. Using the structure of *P. songi* as a reference, CBCs are marked with filled arrows and hemi-CBCs are indicated with hollow arrows. The variable regions are marked by grey boxes. Newly sequenced species are in bold. *, misidentified species.

**Figure 6:**
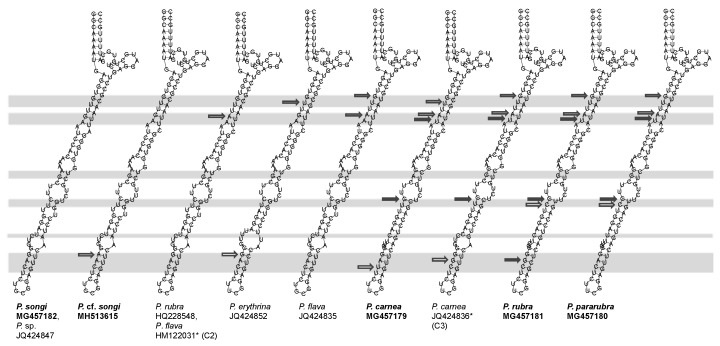
Helix III secondary structures of the *Pseudokeronopsis* LSU-D2 molecule. Using the structure of *P. songi* as a reference, CBCs are marked with filled arrows and hemi-CBCs are indicated with hollow arrows. The variable regions are marked by grey boxes. Newly sequenced species are in bold. *, misidentified species.

The consensus secondary structure of LSU-D2 showed a closed central loop with three helices (I, II, and III; Figure 5A). Helix I was the shortest and most conserved region, which showed no variation among *Pseudokeronopsis* populations (Figure 5A). Helix II contained at least two U-U mismatches and a terminal loop with 5′-AAA versus GGA-3′. Helix III included an A-A/C mismatch and A terminal loop with 5′-AAA versus G-3′, and 5′-UU versus UC (or U) U-3′ (Figure 6). The CBC analysis revealed a maximum of nine CBCs between *Pseudokeronopsis* species and no CBC between the conspecifics (Table 4). 

### 3.3. Molecular Phylogenetic Analyses 

**Figure 7:**
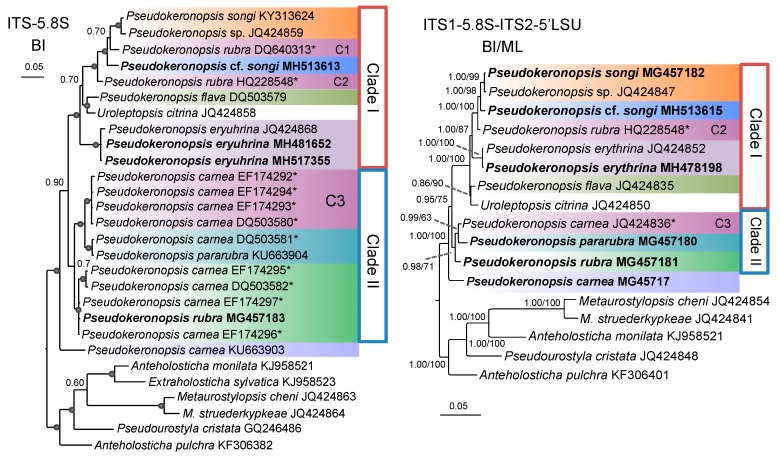
Bayesian inference (BI) trees constructed by ITS1-5.8S-ITS2 (ITS-5.8S) and ITS1-5.8S-ITS2-5′LSU sequences showing phylogenetic relationships among the available pseudokeronopsids. The maximum likelihood (ML) tree and the BI tree of ITS1-5.8S-ITS2-5′LSU are identical in topology, and numbers at the nodes represent BI and ML support values. Newly sequenced species are in bold. *, misidentified species.

The phylogenetic reconstruction of SSU-V4 rDNA showed similar results as that of the nearly full-length SSU rDNA, and the same was true for ITS2 and ITS1-5.8S-ITS2, but with lower resolution for both of the part regions. Hence, the SSU-V4 and ITS2 topologies are not provided here. The COI trees constructed using the nucleotide and the amino acid sequences were identical in topology. Thus, only the nucleotide tree, which had relatively higher support values, was selected for the analyses. Both the Bayesian inference (BI) and maximum likelihood analyses (ML) yielded a nearly identical topology by using either the concatenated ITS1-5.8S-ITS2-5′LSU or the SSU rDNA datasets. Thus, a single topology was presented for each of these datasets with support values indicated near branches (Figure 7; Figure S2). 

**Figure 8:**
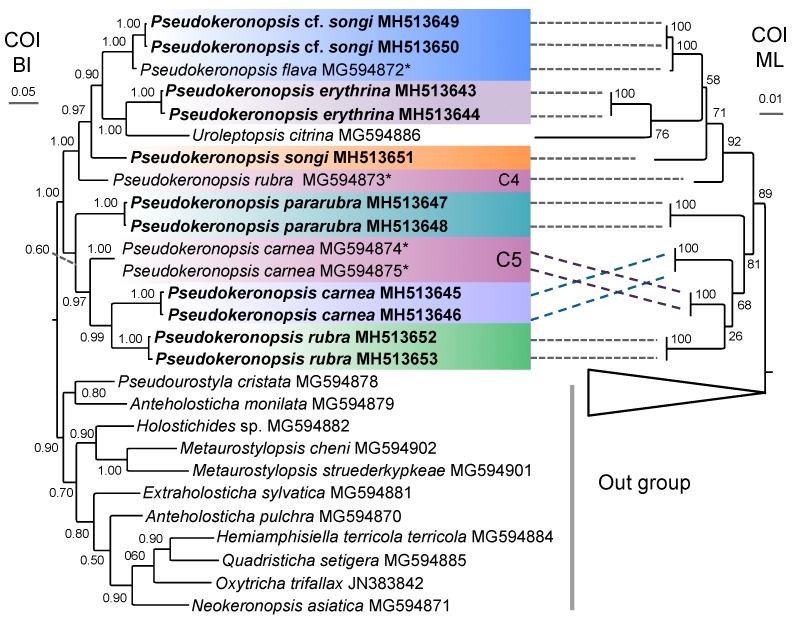
Bayesian inference (BI) and maximum likelihood (ML) trees based on the COI gene showing phylogenetic relationships among the available pseudokeronopsids. Numbers at the nodes represent BI and ML support values. Newly sequenced species are in bold. *, misidentified species.

In all the phylogenetic trees, the branch of *Pseudokeronopsis* consisted of two major clades/groups (I and II; Figure 7 and Figure 8; Figures S2–S4). In sum, Clade I contained *P. songi*, *P.* cf. *songi, P. flava*, *P. erythrina*, *Pseudokeronopsis* sp. JQ424859, three uncertain/cryptic *Pseudokeronopsis* species (marked with C1, C2, C4), and *Uroleptopsis citrina*. Clade/Group II included *P. rubra*, *P. pararubra*, and two uncertain/cryptic species (marked with C3, C5). In all the trees, the genus *Pseudokeronopsis* was not monophyletic due to *Uroleptopsis citrina* nesting into the Clade I with low to high support. 

## 4. Discussion

### 4.1. Suitable Barcodes for Species Delimitation of Pseudokeronopsis

On the basis of the revision of [12,16] and the present study, the genus *Pseudokeronopsis* now contains 12 species, and most of them occur at high frequencies in marine or limnetic habitats worldwide [7,12,16,19,20,58]. However, *Pseudokeronopsis* is still one of the most difficult genera for species discrimination due to their high morphological similarity [12,16,19]. Misidentifications are frequent and thus there is a pressing need to assess DNA barcodes for the species delimitation [12]. 

In the present study, both the ITS1-5.8S-ITS2 and ITS2 regions showed a similar degree of genetic variations and distinct gaps between the intra- and interspecific distances (0.52–3.72% for ITS2; 0.42–3.84% for ITS-5.8S-ITS2), suggesting they are promising barcodes for *Pseudokeronopsis.* Considering that the almost all intraspecific genetic distances of the previously investigated ciliates are no more than 1.5% [14,15,25,26,30,31,32,43,59,60], we identified a sequence divergence of 1.5% as an ideal threshold of ITS1-5.8S-ITS2 and ITS2 to discriminate *Pseudokeronopsis* species. Like in the case of the ITS region markers, the LSU-D2 showed a distinct genetic distance gap (0–2.1%) as well, indicating it is also a promising barcode. Furthermore, the species delimitation results based on the ITS1-5.8S-ITS2, ITS2, and LSU-D2 regions were identical (Figure 2). Thus, to promote confidence in uncovering novel species, we proposed the multilocus barcoding marker including ITS-5.8S-ITS2 and LSU-D2 as a better choice.

COI has the highest rate of evolution among these markers, and has been frequently tested for DNA barcoding of ciliates [13,14,26,34,61,62,63]. In the present analyses, COI showed a distinct gap (2.58–13.25%) in discriminating all available species, showing the potential for species delimitation of *Pseudokeronopsis*. However, the exact range of intraspecific genetic distance may not have been fully uncovered because the intraspecific data were obtained from only two populations each of *P.* cf. *songi* and the uncertain species C4. It is worth noting that previous studies indicated that the intraspecific divergence range of COI was highly variable, even within the same genus. For instance, the intraspecific genetic distance range was ≤16.4% in *Frontonia canadensis* and ≤4.8% in *F. sinica* [14]. Thus, it is risky to infer a threshold of COI to uncover novel species before comprehensive studies with rich sampling are conducted. 

To summarize, the ITS1-5.8S-ITS2, ITS2, and LSU-D2 regions are promising barcodes for *Pseudokeronopsis*, and the multilocus marker including ITS-5.8S-ITS2 and LSU-D2 is more suitable to uncover novel species. The mitochondrial COI is also a candidate barcode, but more intraspecific data are needed to infer the threshold for species discrimination. In contrast, SSU-V4 is useless in discriminating *Pseudokeronopsis* species due to the absence of a barcoding gap.

### 4.2. Appropriate Metabarcoding Markers for Ciliate Diversity Estimation

On the basis of second-generation sequencing (SGS) methods, the SSU-V4 marker has been widely used for estimating ciliate environmental diversity, with the sequence similarity of 97% used as a threshold to identify species [8,10,64]. In our test, the interspecific genetic distances of *Pseudokeronopsis* were in the range of 0–2.75%, corresponding to 0–6 variable sites and 97.3–100% sequence similarities. Thus, only one and three OTUs/species could be detected from the seven known species when using the SSU-V4 sequence similarity of 97% and 99%, respectively, as a threshold (Table 1; Figure 2). Similar results were obtained in Tintinnida and *Frontonia* [9,14]. As such, using the SSU-V4 sequence similarity of 97% or even 99% as a threshold to delineate species may underestimate the environmental diversity of ciliates. An appropriate marker is thus in pressing need for the estimation of environmental diversity. 

The mitochondrial COI showed potential in discriminating *Pseudokeronopsis* species and several oligohymenophorean taxa, for example, with *Tetrahymena*, *Paramecium*, and *Frontonia* [14,25,34]. However, COI is not a good metabarcode for estimating the ciliate environmental diversity due to several issues. First, the barcoding gaps of COI are highly variable (e.g., about 1–2% in *Tetrahymena* vs. 16.4–25.2% in *Frontonia*) [14,34], making it potentially unable to generalize for different taxa. Second, the lack of universal primer sets hampers its utility as a metabarcode. Additionally, there are no mitochondria in some ecologically important taxa [7]. 

The D1-D2 region (>600 bp) of LSU rDNA has also been proposed as an excellent candidate barcode for ciliates due to the presence of barcoding gaps in *Paramecium* and Tintinnida [5,9]. Considering the second-generation sequencing (SGS) read limitation (<600 bp), Zhao et al. [14] tested the barcoding potentials of the D1 and D2 regions and indicated that only LSU-D2 showed a distinct barcoding gap (2–4.5%) in discriminating *Frontonia* species. In the present study, LSU-D2 also showed a barcoding gap (0–2.1%) to separate congeners. However, the overlapping part (2–2.1%) between the two gaps were too tiny to set a uniform threshold to discriminate all ciliates. On the other hand, the universal primers for the LSU-D2 amplification (<600 bp) were not available, hampering its utility as a SGS metabarcode.

**Figure 9:**
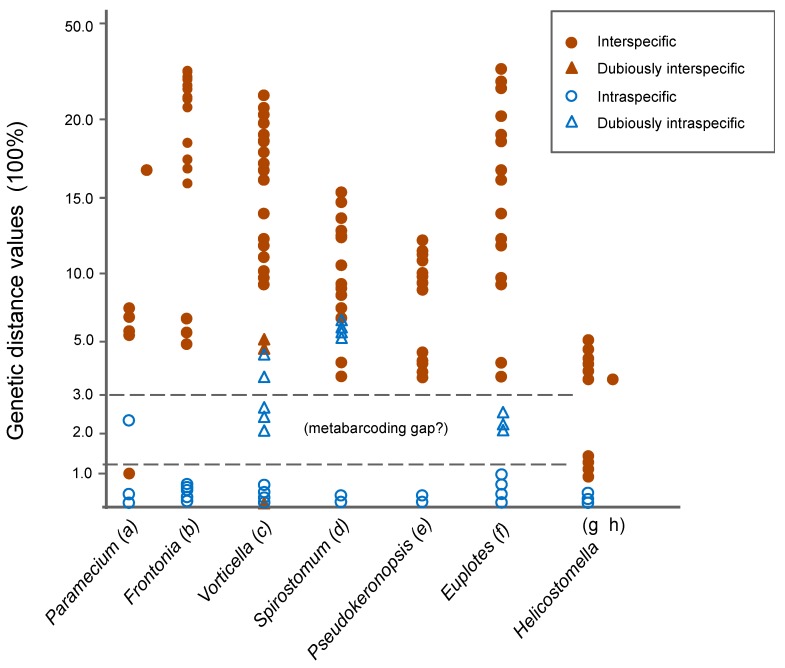
Genetic distances of ITS1-5.8S-ITS2 from the previous and present studies. Data were obtained from or calculated on the basis of the database of (**a**) [25], (**b**) [14], (**c**) [31], (**d**) [30], (**e**) present study, (**f**) [15], (**g**) [60] and (**h**) [33].

The ITS1-5.8S-ITS2 region (<600 bp), by contrast, showed relatively low intraspecific distances (<1.5%) in most ciliate groups investigated, and over half of conspecific sequences were identical even in populations from distant localities (Figure 9) ([14,15,25,26,28,29,30,31,32,33,43,59,60,65,66] and the present study). For instance, no variation in the ITS1-5.8S-ITS2 region was observed for nine populations of *Paramecium caudatum* from China, Germany, Italy, and Australia. The same results were found in *Vorticella convallaria* populations from China and the USA, and in 21 strains of *Philasterides dicentrarchi* isolated from 3 different hosts and 11 localities in Korea and Japan [26,32,59]. When the probable misidentifications mentioned in the above studies were excluded, the interspecific distances in almost all these investigated taxa except for *Paramecium* and *Helicostomella* were >3%, showing a general barcoding gap (Figure 9). 

So far, no distance-based marker could discriminate all closely related species in *Paramecium*, and one explanation is that some species complexes, for example, the *P. bursaria* complex as well as *P. multimicronucelatum* complex, have been officially described as a single species [5,26,66]. The tintinnid *Helicostomella* is a difficult genus possessing polymorphism, making it difficult in unambiguously identifying it down to a species on the basis of lorica morphology, and no single marker (<600 bp) alone can discriminate all the closely related species [33,60]. Therefore, considering the barcoding efficacy and suitable amplication length, we proposed the ITS1-5.8S-ITS2, instead of SSU-V4, as a candidate metabarcode for assessing ciliate environmental diversity. The ITS1-5.8S-ITS2 as a DNA metabarcoding marker can also be complemented by the CBC-based and tree-based criterions (see the following Section 4.3 and Section 4.4). Nevertheless, more genus data from different classes including rich congeneric and conspecific sequences are needed to confirm or reject this hypothesis.

It is worth noting that the third-generation sequencing has been used to study prokaryotic and eukaryotic microbial diversity [67,68]. Longer sequencing reads of several thousand base pairs (bp) are now possible. Considering that the combination of ITS1-5.8S-ITS2 and LSU-D1-D2 performed better than each marker in discriminating closely related species of *Paramecium* and tintinnids [60], the multilocus barcode including ITS1-5.8S-ITS2 and LSU-D2 may provide better taxonomic resolution. The present marker ITS1-5.8S-ITS2-5′LSU (ca. 1300 bp) including the complete ITS-5.8S-ITS2 and LSU-D2 regions is easily amplified in ciliates using the conserved primers ITSF and CILI28S-1000 [40,41], making multilocus barcoding feasible in identifying environmental ciliates with the third-generation sequencing platform. Furthermore, the multilocus metabarcoding marker can also complement the CBC-based and tree-based criteria (see the following Section 4.3 and Section 4.4).

### 4.3. The Species Delimitation Efficiency of CBCs in Putative Secondary Structures

Compensatory base changes (CBCs) in the ITS2 secondary structure have been found to correlate strongly with distinct biological species, and have already been used as a marker for species delimitation in many eukaryotes [43,56,69,70,71,72,73]. For plants and fungi, at least one CBC is a classifier with a 93% reliability that indicates two organisms belonging to two species [70]. This criterion has been tested for the species identification of three ciliate genera, whereas the results are discordant—it worked well for *Paramecium* and *Vorticella* [31,32,43] but was regarded as useless for *Spirostomum* [30]. Whether the CBC-based marker is efficient for ciliate identification in the same way as the corresponding distance-based barcode requires further evaluation. 

In the case of *Pseudokeronopsis*, no CBC was observed between the conspecifics and between a few closely related species, and CBCs only occur between different species (Table 3; Figure 3C and Figure 4). Consequently, the presence of CBCs can be a classifier with a 100% reliability for *Pseudokeronopsis* to assign different populations to separate species. However, the suggested threshold is unidirectional, that is, the lack of CBCs does not necessarily indicate two organisms belonging to the same species, for instance, no CBC occurs between *P. rubra* and *P. pararubra* (Table 3; Figure 3C and Figure 4). This was also shown in *Spirostomum*, *Paramecium,* and *Vorticella*, in which CBCs only exist between distant species, whereas no CBC was observed among some closely related species, for example, *Spirostomum minus*, *S. ambiguum,* and *S. subtilis* [30]. All these test cases indicate that the CBC presence in the ITS2 secondary structure can serve as a good indicator of species separation in ciliates, but not vice versa. 

For those closely related species without CBCs, there are 1–3 hemi-CBCs of ITS2 existing among congeners, whereas 0–1 hemi-CBC occurs among conspecifics (Figure 5C and Figure 6, hollow arrows). Since the taxonomic significance of the presence of hemi-CBCs is still not resolved, more data are needed to test whether a number of ITS2 hemi-CBCs can be served as an indicator of species discrimination within *Pseudokeronopsis*. 

This study is the first attempt to construct a LSU-D2 secondary structure model for a ciliate genus. Among the helices of the model, Helixes II and III contained variant positions to provide cases of CBC and hemi-CBC, the key to “proof” of secondary structure pattern (marked as filled and hollow arrows in Figure 5 and Figure 6). Like the case of ITS2, the presence of one CBC of LSU-D2 can be used separate two organisms into different species with a 100% reliability, whereas the absence of CBCs does not indicate two organisms belonging to the same species. However, due to the conspecific secondary structures being only available from three species of *Pseudokeronopsis*, more conspecific data are needed to confirm this criterion. 

Due to the unidirection of the thresholds, the ITS2 and LSU-D2 CBC-based markers showed lower efficiencies than those of the distance-based barcodes in species delimitation of ciliates. Nevertheless, the CBC-based markers can provide further evidence to separate some closely related species and to uncover cryptic species, for example, one ITS2 CBC occurs between the *songi*-like cryptic species C1 and *P. songi*, and at least one LSU-D2 CBC exits between the *pararubra*-like cryptic species C3 and congeners (Table 3 and Table 4; Figure 3C, Figure 5B and Figure 6). Therefore, the CBC-based marker is useful to complement the traditional DNA barcoding by giving second structure evidence. 

### 4.4. Phylogenetic Relationships within Pseudokeronopsis 

The non-monophyly of *Pseudokeronopsis* was revealed by the rDNA data because *Uroleptopsis citrina* clustered into this group in the SSU, LSU, and ITS-5.8S rDNA trees [24,27,74]. By contrast, in the COI trees (including *U. citrina*) of [27], the *Pseudokeronopsis* species formed a monophyletic clade but with low support. In the present study, *Uroleptopsis citrina* nested into the branch of *Pseudokeronopsis* in both the rDNA and the COI trees with moderate to high support (Figure 7 and Figure 8; Figures S2–S4). However, *Uroleptopsis* and *Pseudokeronopsis* can be easily separated via morphological features, for example, the transverse cirri (absent vs. present) and the position of the buccal cirri (in a pattern of bicorona vs. on the right of paroral) [16,24]. The question whether the morphological differences justify the separation of *U. citrina* and *Pseudokeronopsis* species at genus level has been discussed by [16]. No new sequence of *Uroleptopsis* is available, thus we refrain from this molecular biologically-indicated issue and focus only on the phylogeny of *Pseudokeronopsis*.

The topologies constructed with the selected markers showed more or less differences (Figure 7 and Figure 8; Figures S2–S5). To test which molecule is suitable for elucidating the relationships within *Pseudokeronopsis* properly, we compared the molecular topologies with the morphological data. The SSU rDNA trees formed many parallel clades, which are useless in clarifying the relationships among *P. erythrina*, *P. songi*, *P.* cf. *songi,* and *P. flava* (Figure S2). Likewise, LSU-D2 provides few solutions for this group due to the low support (BI posterior probabilities ≤0.77) for two-thirds of the *Pseudokeronopsis* branches (Figure S3). The ML topology of ITS1-5.8S-ITS2 was also discordant with the morphological data, as *P. pararubra* formed a sister clade with *P. carnea* instead of *P. rubra* (Figure S5). The present COI topologies conflicted with the morphological hypothesis. For example, among the known congeners, *P. songi* is morphologically most similar to *P.* cf. *songi,* but they did not form a sister clade in the COI trees; *P. rubra*, which morphologically resembles *P. pararubra*, fell outside of the clade including *P. pararubra* and *P. carnea* (Figure 8).

**Figure 10:**
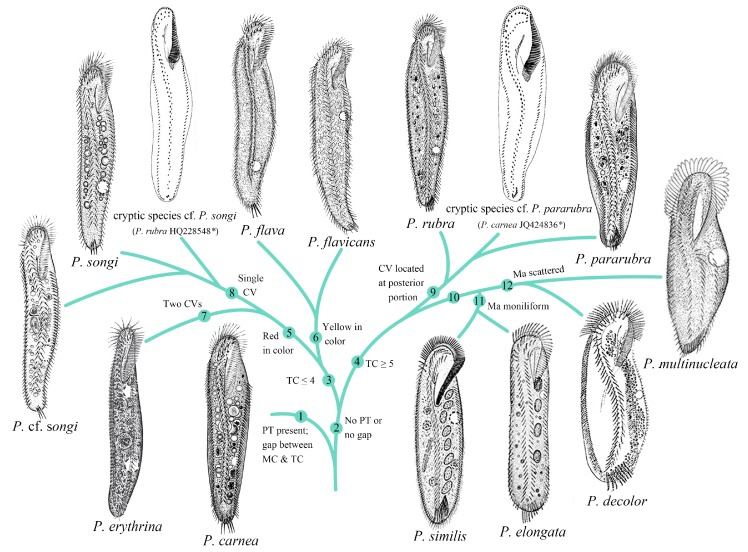
Putative phylogenetic relationships of all available *Pseudokeronopsis* species. *, previously misidentified species. Synapomorphies of major clades were numbered. The number 10 indicates the feature that contractile vacuole is located at mid-body or the anterior portion of body. CV, contractile vacuole; Ma, macronuclear nodules; MC, midventral complex; PT, pretransverse cirri; TC, transverse cirri.

The BI tree of ITS1-5.8S-ITS2, the concatenated ITS1-5.8S-ITS2-5′LSU, and ITS1-5.8S-ITS2-5′LSU-COI trees showed nearly identical topology (Figure 7 and Figure S3), which corresponded well with the morphological data. These markers can provide tree-based evidence to complement the traditional DNA barcoding for ciliate identification. Among these trees, the ITS1-5.8S-ITS2-5′LSU region provided relatively higher support values (nearly all BI posterior probabilities >0.95 and ML bootstraps >70%). Thus, the ITS1-5.8S-ITS2-5′LSU trees were used for elucidating the relationships among *Pseudokeronopsis* species. Within the *Pseudokeronopsis* branch of the ITS1-5.8S-ITS2-5′LSU trees, *P. carnea* branched early, and *P. rubra* and the group *P. pararubra* + the C3 cryptic species formed an intermediate clade (Clade II) between *P. carnea* and the rest of the *Pseudokeronopsis* species (Clade I). Within Clade I, *P. songi* and the C1 cryptic species formed a terminal clade, followed by *P.* cf. *songi,* the uncertain species C2, *P. erythrina,* and *P. flava,* successively. Although the described species *P. multinucleata*, *P. decolor*, *P. elongate,* and *P. similis* have still not been barcoded, their relationships with sequenced taxa can be inferred on the basis of their morphology. These four species are highly similar in ciliary pattern, and close to the Clade II species according to the characters of the midventral complex connected to six or more transverse cirri. On the basis of the position of contractile vacuole, these four species may form a terminal subclade within Clade II (anterior 1/3 or mid-body vs. posterior 1/3–2/5 for the rest species). Within this subclade, *P. decolor* resembles *P. multinucleata* in the position of contractile vacuole (at mid-body), and *P. elongata* is close to *P. similis* due to the distinct moniliform macronucleus and freshwater habitat. Additionally, compared to congeners, the midventral cirral rows of *P. multinucleata* are distinctly separated, also suggesting it nests deeply within Clade II. As such, we combined all available morphological and molecular data and drew a putative evolutionary tree for the genus *Pseudokeronopsis* (Figure 10).

### 4.5. Confirmation and Revision of Pseudokeronopsis Sequences in GenBank

In the nucleotide database of NCBI (https://www.ncbi.nlm.nih.gov/nuccore/?term=txid311385[Organism:exp] on 22 July 2019), there are 140 sequences which were assigned to six known species and four uncertain species of *Pseudokeronopsis.* On the basis of the morphological information and the ITS1-5.8S-ITS2 genetic gap, Li et al. [9] considered that over half of these sequences were misidentified or dubious. However, with the new availability of the ITS1-5.8S-ITS2 sequences of the type species *P. rubra* and *P.* cf. *songi*, we found that the intraspecific genetic distance range sensu [9] is overestimated (0–4.41% vs. the present 0–0.52%). Thus, we confirmed the reliable sequences and revised the misidentified sequences again on the basis of the present genetic distance, secondary structure, and phylogenetic tree data, as well as the morphological information. 

The present genetic distances and the phylogenetic analyses confirmed the validity of *P. carnea*, *P. songi*, *P. pararubra*, *P. erythrina,* and *P. flava* sequences, which were submitted with morphological data [9,25,27,31]. The newly sequenced *P.* cf. *songi* might represent a new species, which is indicated by the genetic distances of the ITS region (>1.5% between it and congeners). On the basis of the sequence characters of *P. rubra* combined with morphological analysis (not shown here), we confirmed that all previously submitted sequences of *P. rubra* were derived from misidentified populations (Table 2).

Without morphological and barcoding data, the identification of the *Pseudokeronopsis* species sensu [30,54] are in progress (Table 2). Additionally, *P. erythrina* was originally described as an estuarine species, whereas the SSU rDNA sequences *P. erythrina* KX459375 and MG994990 were obtained from a freshwater environment. Thus, other barcodes are needed to confirm their validity (Table 2) [22], Kaur et al., unpublished. In sum, a total of 25 misidentified or uncertain *Pseudokeronopsis* sequences were clarified and proposed to be amended, and 65 sequences are still awaiting to be confirmed (Table 2). 

## 5. Conclusions

In the present analyses, all the test markers except SSU-V4 were useful in discriminating *Pseudokeronopsis* species. SSU-V4 rDNA was too conservative for the species discrimination, and only one and three OTUs could be obtained from seven *Pseudokeronopsis* species by using a sequence similarity of 97% and 99%, respectively, as a threshold. The nuclear markers ITS1-5.8S-ITS2, ITS2, and LSU-D2 are promising barcodes, and the multilocus marker including ITS-5.8S-ITS2 and LSU-D2 was more suitable in uncovering novel species. The mitochondrial COI was also a candidate barcode, but more intraspecific data are needed to confirm it. The presence of CBCs was an indicator for *Pseudokeronopsis* to assign different populations to separate species, but not vice versa. The ITS1-5.8S-ITS2-5′LSU region provided better phylogenetic resolution of *Pseudokeronopsis* at species level. At least 25 *Pseudokeronopsis* sequences are misidentified in GenBank and are proposed to be amended. Considering the overlapping barcoding gap between different ciliate taxa, universal primers, suitable amplification lengths, and regularly updated databases available, we propose the multilocus marker including the ITS1-5.8S-ITS2 and LSU-D2 as an ideal candidate barcode, and the ITS1-5.8S-ITS2 as a candidate SGS metabarcode for assessing ciliate environmental diversity. Furthermore, the CBC-based and tree-based criteria are useful to complement the DNA metabarcoding method by giving second structure and phylogenetic evidences. With the application of third-generation sequencing, the multilocus metabarcode may provide greater taxonomic resolution. 

## Figures and Tables

**Table 1:** Specifications of the *Pseudokeronopsis* spp. in the present research. Newly obtained sequences are in bold.

Organisms	Sampling Location	References	Accession Numbers			
			SSU rDNA	ITS1-5.8S-ITS2	LSU rDNA	COI
***Pseudokeronopsis rubra***	Yellow Sea, Qingdao, China	Present study	**MG457184**	**MG457183**	**MG457181**	**MH513652**, **MH513653**
***Pseudokeronopsis pararubra***	Yellow Sea, Qingdao, China	Present study	KU663902	KU663904	**MG457180**	**MH513647**, **MH513648**
***Pseudokeronopsis carnea***	Yellow Sea, Qingdao, China	Present study	KU663901	KU663903	**MG457179**	**MH513645**, **MH513646**
*P. carnea* * (C5)	Yellow Sea, Incheon harbor, Korea	[27,35]	JN714476	-	-	MG594874
*P. carnea* * (C5)	Yellow Sea, Gwangyang, Korea	[27]	MG603616	-	-	MG594875
*P. carnea* *	Yellow Sea, Qingdao, China	[23]	KT984168	-	-	-
*P. carnea* pop-I * (C3)	Yellow Sea, Qingdao, China	[20]	AY881633	DQ503580, EF174292-4	JQ424836	-
*P. carnea* pop-II *	Yellow Sea, Qingdao, China	[20]	-	DQ503581	-	-
*P. carnea* pop-III *	Yellow Sea, Qingdao, China	[20]	-	DQ503582, EF174295-7	-	-
***Pseudokeronopsis erythrina***	Estuary, Yancheng, China	Present study	**MH478194**	**MH481652**, **MH517355**	**MH478198**, **MH478200**	**MH513643**, **MH513644**
*P. erythrina*	Estuary of Pearl River, Guangzhou, China	[18,24]	FJ775723	JQ424868	JQ424852	-
*P. erythrina*	Estuary of Pearl River, Guangzhou, China	[23]	KT984173	-	-	-
*P. erythrina*	Freshwater River Yamuna, Delhi, India	Kaur et al., unpublished	MG994990	-	-	-
*P. erythrina*	Lake Trasimeno, Perugia, Italy	[22]	KX459375	-	-	-
***Pseudokeronopsis* cf. *songi***	Yellow Sea, Qingdao, China	Present study	**MH513618**, **MH513619**	**MH513613**, **MH513614**	**MH513615**, **MH513616**	**MH513649**, **MH513650**
***Pseudokeronopsis songi***	Yellow Sea, Qingdao, China	Present study	KY313623	KY313624	**MG457182**	**MH513651**
*P. rubra* * (C4)	Sea of Japan, Hwajinpo, Korea	[27]	MG603620		-	MG594873
*P. rubra* * (C1)	Yellow Sea, Qingdao, China	[20]	DQ640314	DQ640313	-	-
*P. rubra* *	-	Khan and Shin, unpublished	HM140387	-	-	-
*P. rubra* *	-	[36]	EF535729	-	-	-
*P. rubra* *	Yellow Sea, Qingdao, China	[27]	KT984169	-	-	-
*P. rubra* *	South China Sea, Guangdong, China	[27]	KT984170	-	-	-
*P. rubra* *	Daya Bay, Guangdong, China	[27]	KT984171	-	-	-
*P. rubra* *	Mangrove wetland, Shenzhen, China	[27]	KT984172	-	-	-
*P. rubra* * (C2)	Yellow Sea, Incheon, Korea	Jung et al., unpublished	HQ228548	HQ228548	HQ228548	-
*Pseudokeronopsis flava*	Sea of Japan, Pohang-is, Korea	[27]	MG603616	-	-	MG594872
*P. flava*	South China Sea, Zhanjiang, China	[20]	AY881634	DQ503579	JQ424835	-
*P. flava*	Yellow Sea, Qingdao, China	[20]	DQ227798	-	-	-
*P. flava*	Mangrove wetland, Shenzhen, China	[27]	KT984174	-	-	-
*P. flava*	Yellow Sea, Qingdao, China	[27]	KT984175	-	-	-
*P. flava*	-	Khan and Shin, unpublished	HM140386	-	HM122031	-
*Pseudokeronopsis* sp.	Yellow Sea, Qingdao, China	[24]	JQ424830	JQ424859	JQ424847	-
*Pseudokeronopsis* sp.	Horniman Museum and Gardens Aquarium, London, England	[37]	KP793002	-	-	-

* Misidentified or dubious species. C1–5 correspond to the cryptic species (C1) related to *P. songi*, the cryptic species (C3) related to *P. pararubra,* and the uncertain species (C2, C4, and C5) in Table 2 and the following phylogenetic trees. SSU rDNA: small subunit ribosomal DNA, LSU rDNA: large subunit ribosomal DNA, COI: cytochrome c oxidase subunit 1.

**Table 2:** Emendation for the misidentified or uncertain *Pseudokeronopsis* sequences in GenBank.

Sequences	Reference	Morphological Data *	Molecular Analyses	Emendation
*P. carnea* DQ503581	[20]	Similar to *P. pararubra* and *P. rubra*	No difference from *P. pararubra* in ITS1-5.8S-ITS2.	*P. pararubra*
*P. carnea* DQ503582, EF174295-7	[20]	Similar to *P. pararubra* and *P. rubra*	No difference from *P. rubra* in ITS2 sequence.	*P. rubra*
*P. carnea* DQ503580, EF174292-4, JQ424836, AY881633 (C3)	[20]	Similar to *P. pararubra* and *P. rubra*	The ITS1-5.8S-ITS2 genetic distances between C3 and congeners were in the range of 3.23–10.74%, similar to those among congeners (3.68–13.01%), indicating a cryptic species.	Cryptic species related to *P. pararubra*
*P. rubra* DQ640313, DQ640314 (C1)	[20]	Similar to *P. songi*	The ITS1-5.8S-ITS2 genetic distances between C1 and congeners were in the range of 3.46–11.93%, indicating a cryptic species.	Cryptic species related to *P. songi*
*P. rubra* MG603620, MG594873 (C4)	[27]	Not available	The genetic distances between C4 and congeners were in the range of 13.55–21.54%, indicating an uncertain or cryptic species.	Misidentified, uncertain species
*P. flava* MG594872, MG603616	[27]	Not available	The COI genetic distance between *P. flava* MG594872 and *P.* cf. *songi* was 2.58%, whereas those among congeners ranged from 13.25% to 21.28%, indicating it conspecific with *P.* cf. *songi*.	*P.* cf. *songi*
*Pseudokeronopsis* sp. JQ424859, JQ424830	[24]	Not available	The ITS1-5.8S-ITS2 genetic distance between JQ424859 and *P. songi* was 0.21% (vs. conspecific distances 0–0.42%), indicating that the former belonged to *P. songi.*	*P. songi*
*P. carnea* JN714476, MG603616, MG594874, MG594875 (C5)	[27]	Not available	The COI genetic distance between *P. carnea* MG594874 and MG594875 was zero, whereas those between them and congeners were much higher than conspecific ones (13.55–20.85% vs. 0–2.58%), suggesting they represented an uncertain species.	Misidentified, uncertain species
*P. rubra* HQ228548 (C2), *P. flava* HM122031	Jung et al. (unpublished), Khan & Shin (unpublished)	Not available	The ITS1-5.8S-ITS2 genetic distance between C2 and congeners were in the range of 3.67–9.73%, suggesting it represented an uncertain species. *P. flava* HM122031 and C2 were conspecific, possessing the identical LSU-D2 sequence.	Misidentified, uncertain species
*P. carnea* KT002202-07, *P. flava* KT002234-44, KR263877-79, KR263883-89, *P. rubra* HM140387, EF535729, KT984169-72, KT002196-201, KT002222-33, KT002245-48, KR263874-76, KR263880-82, KR263888-91	[23,36]	Not available	Only SSU rDNA and Actin I sequences were available for these populations, showing six, one, and three nucleotide differences of SSU-V4 region from the valid *P. carnea, P. flava,* and *P. rubra*, respectively.	Dubious
*P. carnea* GQ258110	[57]	Not available	Only alpha-tubulin gene sequence was available.	Dubious
*P. erythrina* KX459375, MG994990	[22], Kaur et al. unpublished	Not available	Only SSU rDNA sequences were available, showing 1 and 23 nucleotide differences of SSU rDNA from the valid *P. erythrina*, respectively.	Dubious

The morphological data is shown in Figure 10.

**Table 3:** Compensating base changes of ITS2 between *Pseudokeronopsis* species/populations.

Species/Populations		1	2	3	4	5	6	7	8	9	10
1	*P. pararubra* KU663904, *P. carnea* DQ503581 *	0									
2	*P. carnea* DQ503580, EF174292-7 * (C3)	0	0								
3	*P. carnea* KU663903	1	1	-							
4	*P. songi* KY313624, *Pseudokeronopsis* sp. JQ424859	2	2	2	0						
5	*P. rubra* DQ640313 * (C1)	1	1	1	1	-					
6	***P.* cf. *songi* MH513613, MH513614**	1	1	1	0	0	0				
7	*P. rubra* HQ228548 * (C2)	1	1	1	0	0	0	-			
8	*P. flava* DQ503579	2	1	1	2	1	1	0	-		
9	***P. erythrina*MH481652**, **MH517355**, JQ424868	2	1	1	1	0	0	0	1	0	
10	***P. rubra*MG457183**, *P. carnea* DQ503582 *	0	0	1	1	1	0	1	1	1	0

* Misidentified species. C1–3 correspond to the C1–3 taxa in the ITS1-5.8S-ITS2 tree. Newly obtained sequences are in bold.

**Table 4:** Compensating base changes of LSU-D2 between *Pseudokeronopsis* species/populations.

Species/Populations		1	2	3	4	5	6	7	8	9
1	***P. songi***, *Pseudokeronopsis* sp. JQ424847	0								
2	***P.* cf. *songi*MH513615**, **MH513616**	0	0							
3	*P. flava* JQ424835	1	1	-						
4	***P. erythrina*MH478198**, **MH478200**, JQ424852	2	3	0	0					
5	***P. carnea*** **MG457179**	7	8	7	9	-				
6	***P. pararubra*** **MG457180**	5	6	5	8	1	-			
7	***P. rubra*** **MG457181**	7	8	6	8	2	1	-		
8	*P. carnea* JQ424836 * (C3)	5	6	6	7	1	1	1	-	
9	*P. rubra* HQ228548 *, *P. flava* HM122031 (C2)	0	0	0	1	7	5	7	5	0

* Misidentified species. C2–3 correspond to the C2–3 taxa in the ITS1-5.8S-ITS2-5′LSU tree. Newly obtained sequences are in bold.

## Supplementary Materials

The following are available online at https://www.mdpi.com/2076-2607/7/11/493/s1: Figure S1: Alignment of ITS2 showing the lengths of the helices. Figure S2: Bayesian inference (BI) and maximum likelihood (ML) trees based on 18S rDNA sequences showing phylogenetic relationships among the available pseudokeronopsids. Numbers at the nodes represent BI and ML support values. Newly sequenced species are in bold. *, misidentified. Figure S3: Maximum likelihood (ML) and Bayesian inference (BI) trees based on the concatenated ITS1-5.8S-ITS2-5′LSU-COI (ITS-COI) sequences showing phylogenetic relationships among the available pseudokeronopsids. Numbers at the nodes represent ML and BI support values. Newly sequenced species are in bold. *, misidentified. Figure S4: Bayesian inference (BI) tree based on LSU-D2 sequences showing phylogenetic relationships among the available pseudokeronopsids. Numbers at the nodes represent BI support values. Newly sequenced species are in bold. *, misidentified. **, uncertain species. Figure S5: Maximum likelihood (ML) tree based on ITS1-5.8S-ITS2 sequences showing phylogenetic relationships among the available pseudokeronopsids. Numbers at the nodes represent BI support values. Newly sequenced species are in bold. *, misidentified. **, uncertain species. Table S1: Genetic distances of COI between *Pseudokeronopsis* species/populations. Table S2: Genetic distances of ITS1-5.8S-ITS2 between *Pseudokeronopsis* species/populations. Table S3: Genetic distances of ITS2 between *Pseudokeronopsis* species/populations. Table S4 Genetic distances of LSU-D2 between *Pseudokeronopsis* species/populations. Table S5 Genetic distances of SSU-V4 between *Pseudokeronopsis* species/populations.
