# Supplementary material for: Ciliate Environmental Diversity Can Be Underestimated by the V4 Region of SSU rDNA: Insights from Species Delimitation and Multilocus Phylogeny of Pseudokeronopsis (Protist, Ciliophora)
Source: Microorganisms. 2019 Oct 26;7(11):493. doi: 10.3390/microorganisms7110493 (PMC6920991; doi:10.3390/microorganisms7110493)
Supplement: Supplementary file 1 [file microorganisms-07-00493-s001.pdf]

Table S1 Genetic distances of COI between *Pseudokeronopsis* species/populations.

	species/populations	1	2	3	4	5	6	7	8	9
1	***P. songi*** **MH513651**	–								
2	***P.*** **cf.** ***songi*** **MH513649–50**	14.27%	0							
3	***P. erythrina*** **MH513643–4**	17.34%	18.16%	0						
4	***P. carnea*** **MH513645–6**	20.47%	21.83%	22.27%	0					
5	***P. pararubra*** **MH513647–8**	19.52%	19.55%	22.04%	20.15%	0				
6	***P. rubra*** **MH513652–3**	21.18%	20.99%	21.14%	18.20%	19.81%	0			
7	*P. flava* MG594872	13.25%	2.58%	16.30%	19.54%	19.83%	21.28%	–		
8	*P. carnea* MG594874–5* (C5)	16.56%	18.21%	20.85%	15.53%	17.08%	16.09%	17.12%	0	
9	*P. rubra* MG594873* (C4)	14.00%	13.80%	17.35%	21.54%	18.21%	17.38%	13.55%	16.06%	–

*Misidentified species. C4–5 correspond to the C4–5 taxa in the COI tree. Newly obtained sequences are in bold. The data marked with gray shadows were excluded from the initial statistics.

Table S2 Genetic distances of ITS1-5.8S-ITS2 between *Pseudokeronopsis* species/populations.

	species/populations	1	2	3	4	5	6	7	8	9	10	11	12	13	14
1	*P. songi* KY313624	–													
2	***P.* cf. *songi* MH513613–4**	4.13%	–												
3	*P. flava* DQ503579	8.01%	7.07%	–											
4	*P. erythrina* JQ424868	9.73%	8.50%	6.60%	–										
5	***P. erythrina* MH481652, MH517355**	9.23%	8.02%	6.14%	0.42%	0									
6	*P. carnea* KU663903	13.01%	12.20%	10.42%	12.44%	11.93%	–								
7	*P. pararubra* KU663904, *P. carnea* DQ503581	10.71%	9.00%	7.78%	7.07%	6.61%	8.95%	0							
8	***P. rubra* MG457183**, *P. carnea* EF174296–7	10.27%	9.02%	7.78%	8.04%	7.56%	8.26%	3.68%	0						
9	*Pseudokeronopsis* sp. JQ424859	0.21%	4.35%	8.25%	9.97%	9.48%	13.28%	10.97%	10.52%	–					
10	*P. rubra* DQ640313[*] (C1)	3.46%	3.67%	7.53%	8.96%	8.48%	11.93%	9.46%	9.48%	3.68%	–				
11	*P. rubra* HQ228548[*](C2)	4.36%	3.91%	5.24%	7.83%	7.35%	9.49%	7.82%	7.84%	4.59%	3.67%	–			
12	*P. carnea* EF174292[*] (C3)	10.74%	10.49%	8.77%	8.99%	8.50%	8.99%	3.45%	4.57%	10.99%	9.73%	7.84%	–		
13	*P. carnea* DQ503580, EF174293–4[*] (C3)	10.49%	10.24%	8.52%	8.75%	8.26%	8.75%	3.23%	4.34%	10.74%	9.48%	7.60%	0.21%	0	
14	*P. carnea* EF174295, DQ503582[*]	10.50%	9.26%	8.02%	8.27%	7.79%	8.50%	3.90%	0.21%	10.76%	9.72%	8.07%	4.79%	4.57%	0

[*]Misidentified species. C1–3 correspond to the C1–3 taxa in the ITS1-5.8S-ITS2 tree. Newly obtained sequences are in bold. The data marked with gray shadows were excluded from the initial statistics.

Table S3 Genetic distances of ITS2 between *Pseudokeronopsis* species/populations.

	species/populations	1	2	3	4	5	6	7	8	9	10	11	12
1	*P. songi* KY313624	–											
2	***P.* cf. *songi* MH513613, MH513614**	6.55%	0										
3	*P. flava* DQ503579	11.23%	8.82%	–									
4	*P. erythrina* JQ424868	13.73%	10.00%	9.37%	–								
5	***P. erythrina* MH481652, MH517355**	13.09%	9.40%	8.79%	0.52%	0							
6	*P. carnea* KU663903	15.28%	13.88%	13.03%	16.93%	16.26%	–						
7	*P. pararubra* KU663904, *P. carnea* DQ503581	15.10%	11.26%	8.84%	9.38%	8.79%	13.67%	0					
8	***P. rubra* MG457183**, *P. carnea* DQ503582, EF174295–7	15.97%	13.22%	10.64%	13.13%	12.49%	12.57%	7.67%	0				
9	*Pseudokeronopsis* sp. JQ424859	0.52%	7.13%	11.85%	14.39%	13.73%	15.97%	15.78%	16.67%	–			
10	*P. rubra* DQ640313[*] (C1)	4.83%	4.85%	7.63%	9.97%	9.37%	11.85%	10.59%	12.49%	5.40%	–		
11	*P. rubra* HQ228548[*] (C2)	5.99%	5.44%	5.93%	10.02%	9.42%	8.87%	8.84%	10.71%	6.57%	3.72%	–	
12	*P. carnea* DQ503580, EF174292–4[*] (C3)	15.10%	12.52%	10.05%	10.56%	9.96%	11.20%	5.39%	6.51%	15.78%	11.20%	8.84%	0

[*]Misidentified species. C1–3 correspond to the C1–3 taxa in the ITS1-5.8S-ITS2 tree. Newly obtained sequences are in bold. The data marked with gray shadows were excluded from the initial statistics.

Table S4 Genetic distances of LSU-D2 between *Pseudokeronopsis* species/populations.

	species/populations	1	2	3	4	5	6	7	8	9
1	***P. songi***, *Pseudokeronopsis* sp. JQ424847	0								
2	***P.* cf. *songi* MH513615, MH513616**	2.53%	0							
3	*P. flava* JQ424835	3.84%	4.75%	–						
4	***P. erythrina* MH478198, MH478200,** JQ424852	5.58%	5.59%	5.14%	0					

5	***P. carnea* MG457179**	15.20%	16.86%	15.81%	16.74%	–				
6	***P. pararubra* MG457180**	13.18%	14.25%	11.71%	14.67%	6.99%	–			
7	***P. rubra* MG457181**	15.27%	17.51%	13.77%	16.81%	7.93%	2.54%	–		
8	*P. carnea* JQ424836* (C3)	13.77%	14.87%	12.73%	16.36%	7.42%	3.84%	4.73%	–	
9	*P. rubra* HQ228548, *P. flava* HM122031* (C2)	2.10%	2.11%	2.53%	5.14%	15.24%	12.70%	14.78%	13.28%	0

*Misidentified species. C2–3 correspond to the C2–3 taxa in the LSU-D2 tree. Newly obtained sequences are in bold. The data marked with gray shadows were excluded from the initial statistics.

Table S5 Genetic distances of SSU-V4 between *Pseudokeronopsis* species/populations.

	species/populations	1	2	3	4	5	6	7	8	9
1	***P.* cf. *songi* MH513618–9**, *P. songi* HY313632	0								
2	*P. flava* DQ227798, AY881634	0.0045	0							
3	***P. erythrina* MH478194**, FJ775723, KT984173, MG994990, KX459375	0.0091	0.0136	0						
4	*P. carnea* KU663901	0.0091	0.0136	0.0183	–					
5	*P. pararubra* KU663902, *P. carnea* KT984168*	0.0182	0.0228	0.0182	0.0275	0				
6	***P. rubra* MG457184**	0.0136	0.0182	0.0228	0.0228	0.0045	–			
7	*P. rubra* DQ640314 (C1), EF535729*	0.0090	0.0136	0.0182	0.0182	0.0274	0.0228	0		
8	*P. carnea* AY881633 (C5), MG603616, JN714476* (C3)	0.0136	0.0182	0.0136	0.0229	0.0045	0.0090	0.0228	0	
9	*Pseudokeronopsis* sp. JQ424830, KP793002, *P. rubra* HM140387, MG603620 (C4), HQ228548 (C2), KT984169–72*, *P. flava* HM140386, MG603597, KT984174–5	0.0000	0.0045	0.0091	0.0091	0.0182	0.0136	0.0090	0.0136	0

*Misidentified species. C1–5 correspond to the C1–5 taxa in the SSU rDNA trees. Newly obtained sequences are in bold. The data marked with gray shadows were excluded from the initial statistics.

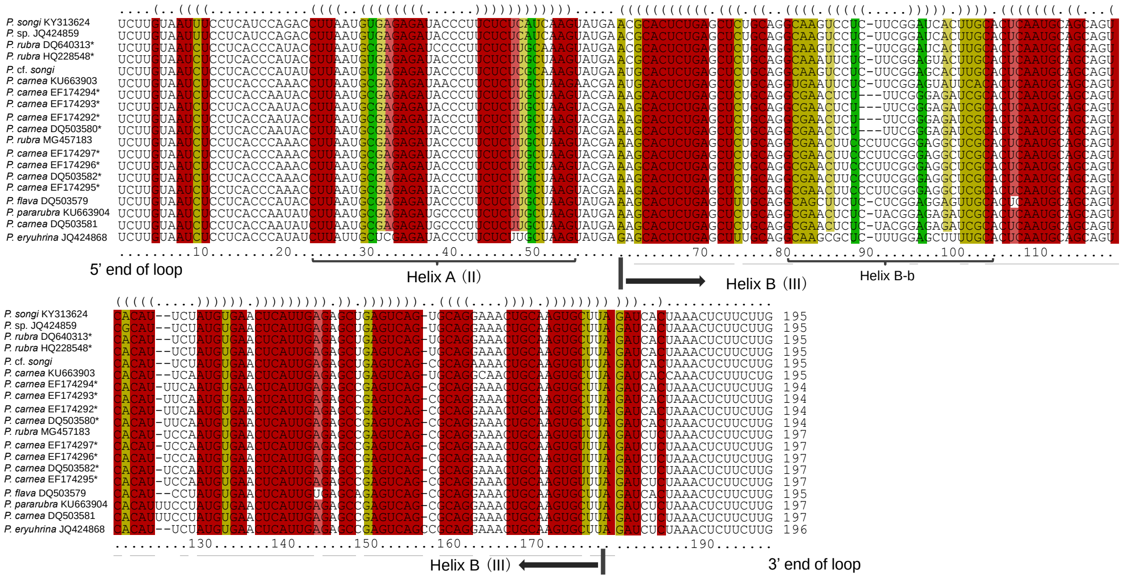

Figure S1. Alignment of ITS2 showing the lengths of the helices.

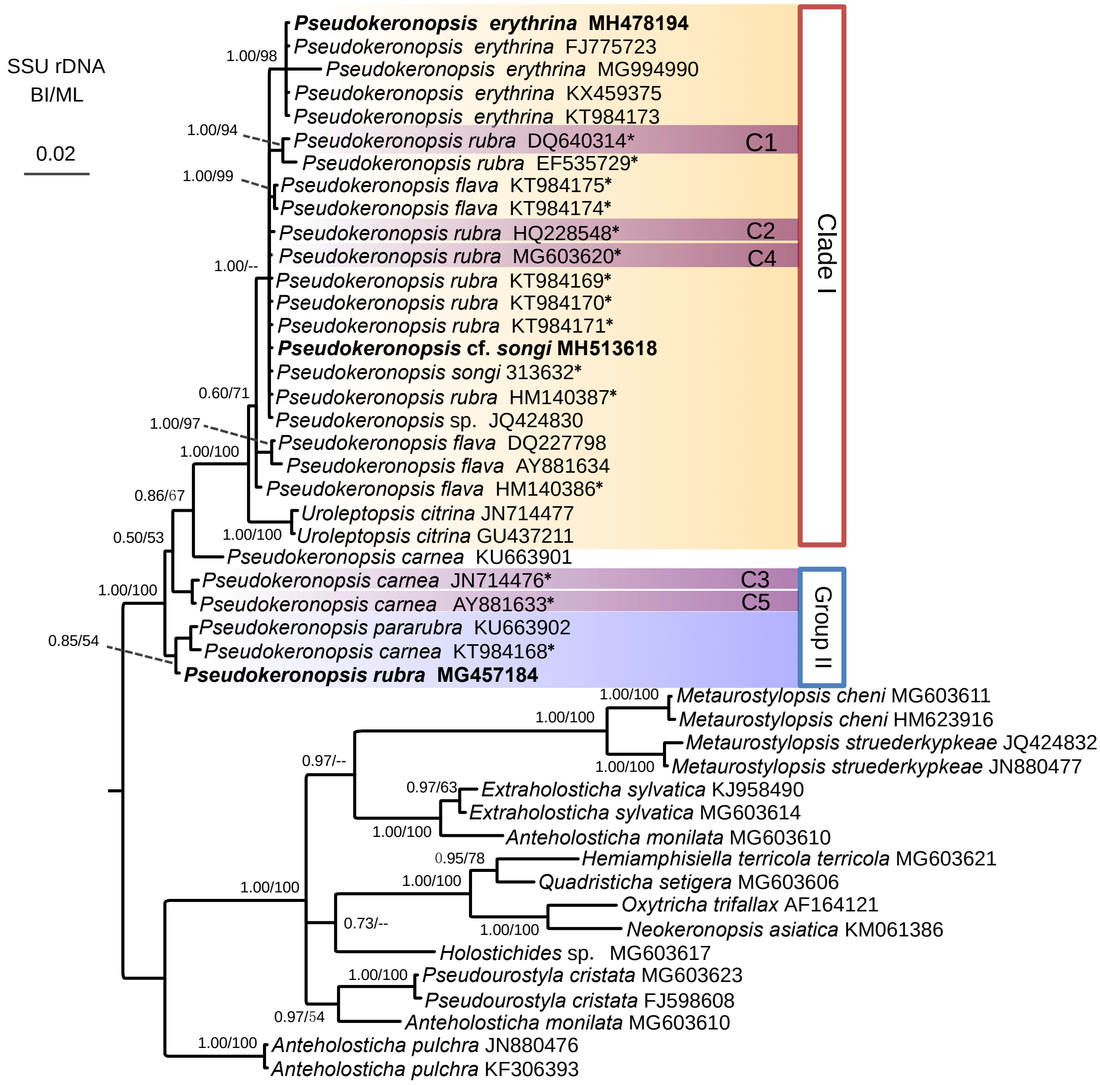

Figure S2. Bayesian inference (BI) and maximum likelihood (ML) trees based on 18S rDNA sequences showing phylogenetic relationships among the available pseudokeronopsids. Numbers at the nodes represent BI and ML support values, respectively. Newly sequenced species are in bold. *, misidentified.

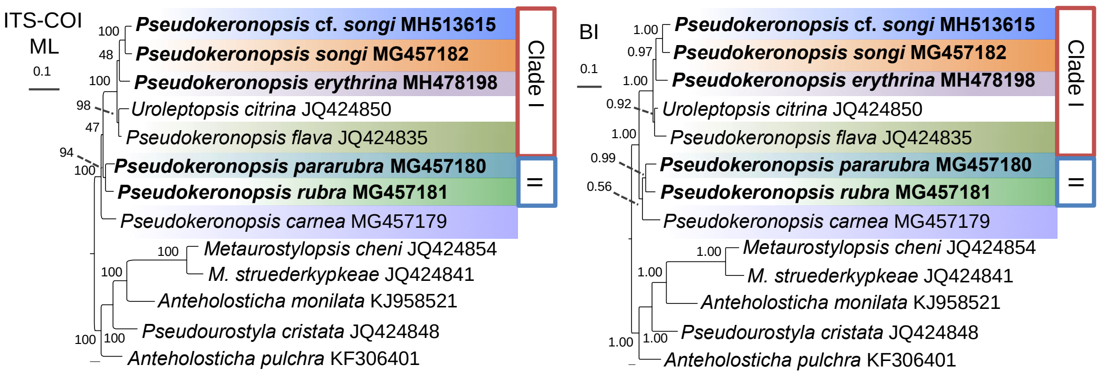

Figure S3. Maximum likelihood (ML) and Bayesian inference (BI) trees based on the concatenated ITS1-5.8S-ITS2-5′LSU-COI (ITS-COI) sequences showing phylogenetic relationships among the available pseudokeronopsids. Numbers at the nodes represent ML and BI support values, respectively. Newly sequenced species are in bold. *, misidentified.

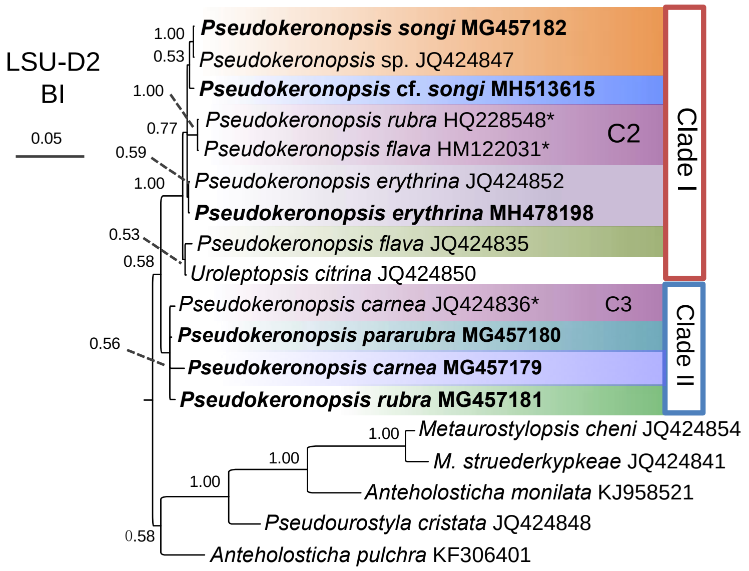

Figure S4. Bayesian inference (BI) tree based on LSU-D2 sequences showing phylogenetic relationships among the available pseudokeronopsids. Numbers at the nodes represent BI support values. Newly sequenced species are in bold. *, misidentified.

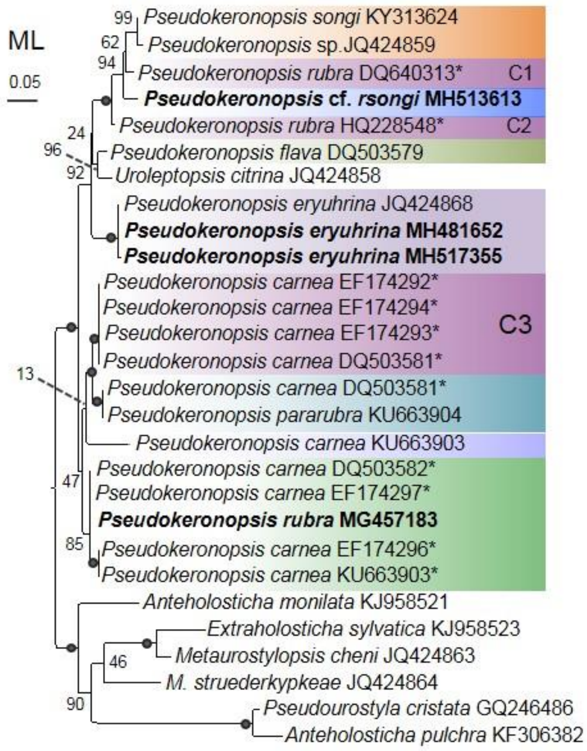

Figure S5. Maximum likelihood (ML) tree based on ITS1-5.8S-ITS2 sequences showing phylogenetic relationships among the available pseudokeronopsids. Numbers at the nodes represent BI support values. Newly sequenced species are in bold. *, misidentified.
